# A Proof-of-Concept Inhibitor of Endothelial Lipase Suppresses Triple-Negative Breast Cancer Cells by Hijacking the Mitochondrial Function

**DOI:** 10.3390/cancers14153763

**Published:** 2022-08-02

**Authors:** Rongze Yang, Shuyan Han, Joseph Clayton, Mahan Haghighatian, Cheng-Chieh Tsai, Yuan Yao, Pingping Li, Jana Shen, Qun Zhou

**Affiliations:** 1 VA Maryland Health Care System, Department of Biochemistry and Molecular Biology, University of Maryland School of Medicine, Baltimore, MD 21201, USA; royang@medicine.umaryland.edu (R.Y.); mhaghigh@terpmail.umd.edu (M.H.); yyao@som.umaryland.edu (Y.Y.); 2 Department of Integration of Chinese and Western Medicine, Key Laboratory of Carcinogenesis and Translational Research (Ministry of Education), Peking University Cancer Hospital & Institute, Beijing 100142, China; shuyanhan@bjmu.edu (S.H.); lppma123@163.com (P.L.); 3 Department of Pharmaceutical Sciences, University of Maryland School of Pharmacy, Baltimore, MD 21201, USA; joseph.clayton@rx.umaryland.edu (J.C.); kevtsai@umaryland.edu (C.-C.T.); jana.shen@rx.umaryland.edu (J.S.); 4 Neuroscience at College of Computer, Mathematical, and Natural Sciences, University of Maryland, College Park, MD 20742, USA

**Keywords:** EL/LIPG, long non-coding RNA DANCR, a new LIPG inhibitor cynaroside, TNBC, metabolism, histone deacetylase HDAC6

## Abstract

**Simple Summary:**

Endothelial lipase (EL/LIPG) is a key regulator of tumor cell metabolism. In triple-negative breast cancer (TNBC) cells, we find that the expression of LIPG is associated with long non-coding RNA DANCR and positively correlates with gene signatures of mitochondrial metabolism-oxidative phosphorylation (OXPHOS). DANCR binds to LIPG, which enables tumor cells to maintain the expression. Importantly, LIPG knockdown inhibits OXPHOS and TNBC tumor formation. Finally, our study identifies a natural compound, the LIPG inhibitor cynaroside, which provides a new therapeutic strategy against TNBC.

**Abstract:**

Triple-negative breast cancer (TNBC) cells reprogram their metabolism to provide metabolic flexibility for tumor cell growth and survival in the tumor microenvironment. While our previous findings indicated that endothelial lipase (EL/LIPG) is a hallmark of TNBC, the precise mechanism through which LIPG instigates TNBC metabolism remains undefined. Here, we report that the expression of LIPG is associated with long non-coding RNA DANCR and positively correlates with gene signatures of mitochondrial metabolism-oxidative phosphorylation (OXPHOS). DANCR binds to LIPG, enabling tumor cells to maintain LIPG protein stability and OXPHOS. As one mechanism of LIPG in the regulation of tumor cell oxidative metabolism, LIPG mediates histone deacetylase 6 (HDAC6) and histone acetylation, which contribute to changes in IL-6 and fatty acid synthesis gene expression. Finally, aided by a relaxed docking approach, we discovered a new LIPG inhibitor, cynaroside, that effectively suppressed the enzyme activity and DANCR in TNBC cells. Treatment with cynaroside inhibited the OXPHOS phenotype of TNBC cells, which severely impaired tumor formation. Taken together, our study provides mechanistic insights into the LIPG modulation of mitochondrial metabolism in TNBC and a proof-of-concept that targeting LIPG is a promising new therapeutic strategy for the treatment of TNBC.

## 1. Introduction

TNBC acquires the increased dependence on OXPHOS during tumor progression [1,2,3]. OXPHOS contributes to drug resistance, recurrence, and mortality in patients with TNBC [4]. Endothelial lipase (EL/LIPG) displays phospholipase A1 activity, which is responsible for lipoprotein metabolism in breast cancer and other types of tumors [5,6]. Several studies have demonstrated that LIPG promotes breast cancer initiation and progression though mediating the metabolism of intracellular lipids [7,8,9,10]. There is increasing evidence that polyunsaturated fatty acids (PUFAs) play a role in tumor progression [11,12]. Supporting this notion, tumor cells preferentially uptake PUFAs, which provide a key substrate to synthesize cell membrane phospholipids for tumor cell proliferation [13]. LIPG overexpression facilitates the uptake of PUFAs, thereby contributing to tumor cell survival [12]. The previous literature has shown that monounsaturated fatty acids have the ability to reduce the risk of breast cancer [14]. A downregulation of LIPG leads to reprogramming lipid metabolism and an inhibition of breast cancer cell growth [7]. These findings suggest a unique role of LIPG in the regulation of lipid metabolism in tumor cells. We previously reported that LIPG prominently expresses in TNBC for tumor metastasis [8]. However, the mechanisms by which LIPG overexpresses in TNBC are not well defined.

Long non-coding RNAs (lncRNAs) are a new class of stem cell regulators that play important roles in a wide range of cellular processes [15]. Studies employing powerful new technologies and bioinformatics tools have identified lncRNAs (with greater than 200 nucleotides in length) that can regulate oncogene expression by binding to chromatin-modifying factors and transcription factors or altering the splicing, stability, or translation of mRNAs [16,17,18,19]. The expression patterns and functions of lncRNAs are highly cell-type specific. LncRNA DANCR is also named anti-differentiation non-coding RNA (ANCR), which is largely presented in the cytoplasmic fraction. DANCR enhances tumor cell proliferation, invasion, and metastasis in TNBC [20]. DANCR can promote tumor progression by inducing the epithelial–mesenchymal transition (EMT) [21]. Furthermore, DANCR contributes to protein assembly and modification, and the activation of the Wnt/β-catenin signaling pathway and the PI3K/AKT pathway, subsequently leading to rapid tumor growth and chemotherapy failure [22,23,24]. During and after transcription, LIPG is subjected to multiple actions that dynamically interact with RNA-binding proteins to regulate its maturation, stability, and functions. Therefore, we need to understand the molecular mechanisms underlying DANCR’s functions in the LIPG signaling pathway. As DANCR is restricted to the cytoplasm, we developed an RNA pulldown method to study DANCR-LIPG interactions in TNBC cells. We demonstrated that DANCR binds to LIPG for the maintenance of LIPG protein expression in TNBC cells. Further, we revealed that the DANCR-LIPG axis can mediate OXPHOS and oleic acid uptake in tumor cells. To translate our findings into a therapeutic approach for breast cancer treatment, we identified a new LIPG inhibitor, cynaroside isolated from the herbal plant dandelion, and confirmed that the inhibition of LIPG is a promising strategy to combat TNBC tumor growth.

## 2. Materials and Methods

### 2.1. Cell Lines and Reagents

MDA-MB-468, MDA-MB-231, and MCF-7 cells were obtained from ATCC (American Type Culture Collection, Manassas, VA, USA). Stable LIPG-overexpression (468-OE) and LIPG-knockdown (468-KD) cell lines were established as described previously [8]. Cells were cultured in DMEM high glucose medium with 5% fetal bovine serum (FBS), 100 U/mL penicillin, and 100 mg/mL streptomycin in a 5% CO_2_ humidified incubator at 37 °C. Cynaroside was purchased from Sigma-Aldrich (St. Louis, MO, USA). The siRNA transfection was performed with 40 nM siRNA using Lipofectamine RNAiMAX (Thermo Fisher Scientific, Waltham, MA, USA) according to the manufacturer’s instructions. siRNAs were obtained from Sigma-Aldrich. The sequences for siRNA LIPG and shRNA LIPG were described in our published studies [8].

### 2.2. The Seahorse XF Analysis and Sphere Formation Assay

The extracellular acidification rate (ECAR) and oxygen consumption rate (OCR) in tumor cells were measured using the Seahorse XF Analyzer (Agilent) as described by the manufacture protocol. Sphere formation assays were performed as previously described [10]. After one week of sphere culture, formed primary spheres were counted under a microscope according to the sphere size criterion (≥100 μm).

### 2.3. Quantitative RT-PCR (qRT-PCR) Analysis and Western Blot

Total RNA of cultured cells was isolated using the Ambion TRIzol reagent (Thermo Fisher Scientific, Halethorpe, MD, USA) according to the manufactures’ instructions. A qRT-PCR analysis of mRNA expression was performed as described previously with normalization to GAPDH or β-actin [8]. The sequence information of the gene primers used in the qRT-PCR experiments is available upon request. For Western blot, equal amounts of protein from cell lysates were separated on 10% SDS-PAGE gels and subsequently transferred to PVDF membranes (Millipore). The primary antibodies against LIPG, β-catenin, and vimentin were obtained from Abcam and Cell Signaling. The membranes were then incubated with HRP-conjugated secondary antibodies, and the protein bands were visualized by ECL reagents (Millipore). The Western blot data were quantified by a densitometric analysis in Image J. The quantitative protein level data were normalized by the β-actin protein levels.

### 2.4. RNA Pulldown Assay

Stable LIPG-overexpressing cells were co-transfected with plasmids: p_NLS_MCP_GFP and pCDNA3-MS2 (negative control) or pCDNA3-MS2_hDANCR. Cells were harvested at 48 h post-transfection using ice-cold polysome lysis buffer (PLB). Supernatants were incubated with an anti-GFP antibody pre-coated with protein A/G magnetic beads. Protein A/G and antibody complexes were separated, and pellets were washed. RNA was extracted using a Direct-zol RNA MiniPrep kit following the manufacturer’s instructions. The expression levels of LIPG were normalized by GAPDH levels and presented as fold changes compared to a MS2-negative control.

### 2.5. Fatty Acid Uptake Assay

Briefly, tumor cells were plated in a 12-well plate at 1 × 10^5^ cells/well the day before the assay in DMEM with 10% FBS. On the day of assay, cells were washed twice with serum-free medium and then serum-starved for 4 h. To perform the assay, serum-free DMEM medium with 3^H^-oleic acid was prepared as follows: First, 6 μL of 3^H^-oleic acid (Perkin Elmer, Waltham, MA, USA, 289001MC, 5 μCi/μL) was added to 6.5 mL of serum-free DMEM and 50 μL of 12.5 mM cold BSA-conjugated oleic acid. Cells were then incubated for 1, 3, 5, and 10 min, respectively, removed from the medium, and washed with ice-cold PBS. Finally, lysate was added to scintillation fluid to count 3^H^ radioactivity. Fatty acid uptake was expressed as 3^H^ activity normalized by the total protein of cell lysate as measured by Coomassie blue using BSA as a reference [25].

### 2.6. Molecular Dynamics Simulations and Docking

A homology model of LIPG was built using the SWISS-model server [26], using the X-ray structure of lipoprotein lipase (PDB ID:6OB0) as a template. The protonated states of LIPG, including the active-site residues Asp193 and His274 were determined using a state-of-the-art pKa prediction tool. The GPU accelerated implicit-solvent based continuous constant pH molecular dynamics with pH replica exchange (REX-CpHMD) [27,28]. For the REX-CpHMD simulations, the protein was represented by the Amber ff14SB force field [29], and the solvent was represented by the generalized Born model GBNeck2. The system was first energy-minimized and briefly equilibrated at 300 K at pH 7.5 before initiating the REX-CpHMD simulations. For the latter, five replicas placed at pH values 6.5 to 8.5 with an interval of 0.5 pH units were simulated until the protonation states were converged (for 5 ns/replica). The replicas adjacent in pH were allowed to exchange conformations every 2 ps.

Following the determination of protonation states, a 300 ns molecular dynamics (MD) simulation was conducted using AMBER20 to relax the homology model and produce structures for docking. The structure was first energy-minimized for 10,000 steps with a harmonic restraint force constant of 100 kcal/mol/Å^2^ on the heavy atoms of the protein. The steepest decent algorithm was used for the first 1000 steps, followed by the conjugate gradient algorithm for the remaining 9000 steps. With the same harmonic restraints on the protein, the system was then heated to 300 K over 10 ns, followed by equilibration, whereby the harmonic force constant was gradually decreased from 10, 7.5, 3, 1, and 0.1 kcal/mol/Å^2^ over 5 ns to 0 kcal/mol/Å^2^ for 5 ns. A 10 Å distance cutoff was used for van der Waals energies. For long-range electrostatics, the particle mesh Ewald method was used, with the real-space cutoff of 10 Å and 1 Å grid spacing. The simulation was performed at a pressure of 1 atm and a temperature of 300 K, controlled by the Monte Carlo barostat and Langevin thermostat, respectively. The total production run lasted 300 ns, and the first 200 ns were discarded. Ten conformational snapshots of LIPG were selected from the last 100 ns, each separated by 10 ns of simulation time. AutoDockTools4 was used to prepare both the selected frames and the compounds identified in dandelion extract [30], while AutoDock4 was used to perform rigid docking with a 30 Å cubic box centered at the midpoint between Ser169, Asp193, and His274. Nine poses were generated for each ligand and LIPG conformation pair; the estimated binding affinities of these poses were then averaged per ligand after discarding any affinity greater than zero.

### 2.7. Statistical Analysis

The statistical analysis of the general experimental datasets was performed by Student’s *t* test. The *p* values of <0.05 were considered significant. Data were analyzed using GraphPad Prism (version 9.0; GraphPad Software, Inc., La Jolla, CA, USA).

## 3. Results

### 3.1. LIPG Is Correlated with DANCR and Signature Genes of OXPHOS in Human TNBC

**Figure 1:**
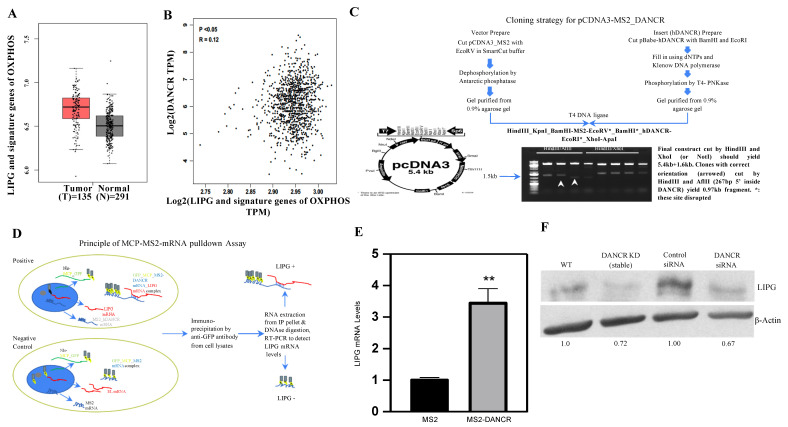
DANCR mediates LIPG expression. (**A**) LIPG and signature genes of OXPHOS in tumor and normal tissues. (**B**) Analysis of correlation between DANCR and LIPG plus signature genes of OXPHOS from IBC tumors. The Pearson method was used for analysis. Correlation is statistically significant (*p* < 0.05). TPM: transcripts per million. (**C**) Cloning strategy for pCDNA3-MS2_DANCR. (**D**) The principle of MCP-MS2-mRNA pulldown assay. (**E**) stable LIPG-overexpressing MDA-MB-468 cells were co-transfected with p_NLS_MCP_GFP and pCDNA3-MS2 (negative control) or pCDNA3-MS2_hDANCR. Cells were collected 48 h after transfection for RNA pulldown assay using anti-GFP antibody. RNA was extracted for qRT-PCR. The expression levels were presented as fold changes compared to MS2 control. ** *p* < 0.01. (**F**) MDA-MB-468 cells were transfected with shRNA DANCR or siRNA DANCR, and protein levels of LIPG were measured by Western blot, the uncropped blots are shown in Figure S5.

Since OXPHOS is a critical metabolism pathway to maintain TNBC tumor growth, we decided to analyze long non-coding RNA DANCR, LIPG, and signature genes of OXPHOS in an RNA-seq database available from the Cancer Genome Atlas (TCGA). Patients with TNBC showed enhanced LIPG and signature genes of OXPHOS (Figure 1A). In patients with invasive breast carcinoma (IBC), higher levels of LIPG and signature genes of OXPHOS were found to be correlated with the presence of DANCR in the tumors (n = 1085, Figure 1B). Together, these data demonstrate that LIPG and DANCR represent two key regulators of OXPHOS metabolism in TNBC. These data provide a rationale for the further investigation of DANCR in the regulation of functional LIPG in tumor cells.

### 3.2. DANCR Maintains LIPG Protein Expression in TNBC Cells

To analyze the interaction between DANCR and LIPG inside tumor cells, we developed an MCP-MS2 system in which GFP-tagged MCP bound to MS2 stem loop sequences that can be immunoprecipitated using antibodies to identify DANCR binding (Figure 1C,D). The human TNBC cell line MDA-MB-468 (hereafter abbreviated 468-WT) with stable overexpression of LIPG (hereafter abbreviated 468-OE) were established [8] and used in the present studies. The 468-OE cells were co-transfected with an MCP-GFP-tagged plasmid with either an MSP-DANCR or MS2 plasmid. GFP was immunoprecipitated with anti-GFP antibodies, and DANCR was purified. Figure 1E shows that LIPG was pulled down 3-fold more in the MS2-DANCR cells than the MS2 cells. The ectopic overexpression of LIPG in luminal breast cancer cell line MCF-7 (hereafter abbreviated MCF7OE) promotes migration, stemness, and basal/EMT features [7]. Similar results were observed in MCF7OE cells (Supplementary Figure S1). To determine a specific DANCR-binding region, pcDNA3-MS2-DANCRwt (1–855 bp) was deleted into DANCR1 (219–855 bp), DANCR2 (429–855 bp), and DANCR3 (639–855 bp). These constructs were transfected into MCF7OE cells (Supplementary Figure S2). An RNA pull-down assay showed that DANCR (1–638 bp) is necessary for the specificity of the interaction of DANCR and LIPG. Collectively, these results suggest that a direct interaction between LIPG and DANCR may impact LIPG expression in tumor cells.

### 3.3. DANCR Knockdown Leads to Downregulation of LIPG Protein

Given that DANCR binds to LIPG in tumor cells (Figure 1E), we next investigated if functional DANCR mediates LIPG expression. The translational control of specific mRNAs has been shown to play a critical role in cancer initiation as well as progression [31]. DANCR knockdown by shRNA does not downregulate LIPG mRNA levels (data not shown). The ribosome-associated mRNA assay provides a powerful method to assess the association of ribosomes with a given mRNA. It provides valuable information about the translational status of a specific mRNA. To determine the impact of DANCR on the ribosomal binding of LIPG mRNA, we efficiently isolated ribosome-associated mRNA transcripts from MDA-MB-468 cells (468-WT) and DANCR knockdown MDA-MB-468 cells and measured ribosome-associated mRNA. Surprisingly, there were similar levels of ribosome-associated LIPG mRNA between 468-WT cells and DANCR knockdown MDA-MB-468 cells (data not shown). Thus, we hypothesized that DANCR may impact LIPG protein expression in tumor cells. The LIPG protein levels in 468-WT cells were dramatically decreased when DANCR was depleted by both shRNA and siRNA (Figure 1F). These data suggest that DANCR interacts with LIPG, which leads to sufficiently maintaining LIPG protein in tumor cells.

### 3.4. LIPG or DANCR Knockdown Mediates Oleic Acid Intake in Tumor Cells

**Figure 2:**
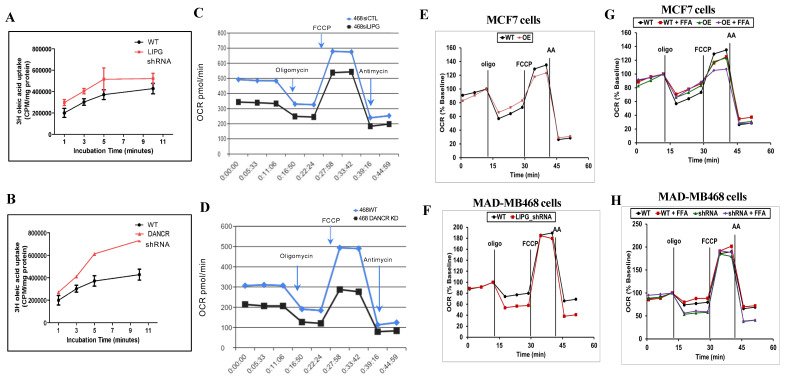
LIPG knockdown inhibits OCR. (**A**,**B**) Oleic acid uptake assay in MDA-MB-468 cells (WT), and LIPG or DANCR shRNA stable MDA-MB-468 cells. (**C**,**D**) Seahorse analysis of oxygen consumption rate (OCR) in wild-type (WT) and LIPG/DANCR siRNA knockdown MDA-MB-468 cells. OCR was measured, and the following drugs were used for the assay: oligomycin (oligo, 0.5 µg/mL); carbonyl cyanide-4 (trifluoromethoxy) phenylhydrazone (FCCP, 4 µM), and antimycin (AA, 1 µM). (**E**,**F**) Seahorse analysis of OCR in wild-type (WT) and LIPG overexpression (OE) MCF-7 or shRNA knockdown (KD) MDA-MB-468 cells. (**G**,**H**) Tumor cells, as indicated, were treated with or without 150 µM FFA, and OCR was subsequently analyzed.

The relationship between oleic acid (OA, monounsaturated fatty acid) and TNBC progression suggests that OA has a protective effect against breast cancer [32]. Treatment with OA inhibits TNBC cell growth [33]. We decided to determine if LIPG may impact OA uptake in tumor cells. 3^H^-radiolabeled OA has the advantage of faithfully mimicking the biochemical properties of natural fatty acids [34]. LIPG shRNA knockdown increased OA uptake in MDA-MB-468 cells by an average of 35% compared with control cells (Figure 2A). Similar results were observed in DANCR knockdown MDA-MB-468 cells (Figure 2B). Since LIPG or DANCR shRNA knockdown leads to the inhibition of tumor cell growth [8,10,23], the findings from Figure 2A,B support that the depletion of LIPG or DANCR enhances OA uptake, which contributes to the suppression of tumor cell growth.

### 3.5. Tumor Cells Rely on LIPG for OXPHOS

To investigate the roles of LIPG in metabolic flexibility, MDA-MB-468 cells were analyzed for their oxygen consumption rate (OCR). LIPG or DANCR siRNA knockdown MDA-MB-468 cells consumed oxygen at significantly lower basal levels, produced much less ATP, and exhibited lower levels of OCR in response to the FCCP, yielding a reduced maximal respiratory capacity (Figure 2C,D). In addition, the knockdown of LIPG or DANCR caused a decrease in mitochondrial spare respiration capacity with a simultaneous reduction in proton leakage. These data suggest that LIPG or DANCR knockdown leads to the suppression of mitochondrial metabolism in TNBC tumor cells.

We previously demonstrated that MCF-7 luminal breast cancer cells express lower levels of LIPG [8]. However, the stable overexpression of LIPG in MCF-7 cells promotes basal/EMT features [8]. To further confirm that LIPG is required for OXPHOS in TNBC cells, we measured OXPHOS in LIPG-overexpressing MCF-7 cells (MCF7OE) and LIPG knockdown MDA-MB-468 cells (468-KD). LIPG overexpression led to increased OXPHOS in MCF7OE cells compared to wild-type MCF-7 cells (Figure 2E). In parallel, we observed that shRNA-mediated LIPG knockdown inhibited OXPHOS in MDA-MB-468 cells (Figure 2F). All these observations indicate that LIPG is a key lipolytic enzyme involved in tumor cell OXPHOS.

Fatty acid (FFA) is an important energy source that promotes tumor cell growth through mitochondrial fatty acid oxidation (FAO) [34]. To fuel FAO for tumor cell growth, LIPG degrades lipoproteins and subsequently releases fatty acids. We therefore investigated if LIPG in tumor cells plays a key role in regulating FAO. LIPG overexpression in MCF7OE cells enhanced FFA-induced OXPHOS compared to FFA-treated wild-type MCF-7 cells (Figure 2G). The absence of LIPG in 468-KD cells specially impaired FFA-induced OXPHOS as compared with FFA-treated 468-WT cells (Figure 2H). In summary, these results demonstrate that tumor cells require LIPG for mitochondrial OXPHOS.

### 3.6. Molecular Mechanisms of LIPG in the Regulation of Tumor Cell Metabolism

Histone acetylation is considered to play significant roles in chromatin accessibility and transcriptional regulation. HDAC6 inhibits the mitochondrial protein PHB1, leading to increased oxidants and oxidative stress in sepsis [35]. Thus, we decided to determine if LIPG participates in remodeling the chromatin structure during tumor cell lipid metabolism.

**Figure 3:**
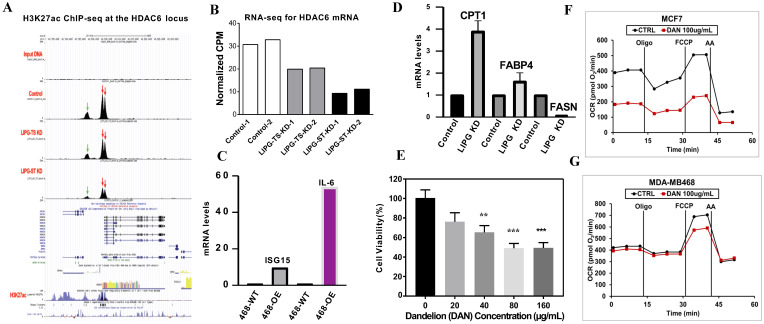
LIPG alters histone acetylation and HDAC6 expression. (**A**) Gene track view for H3K27ac ChIP-seq data at the HDAC6 locus in the indicated cell lines. Two major peaks are located at the promoter region of the HDAC6 gene (indicated by red arrows), and the third peak is located at the distal upstream region (indicated by a green arrow). (**B**) RNA-seq analyses of HDAC6 mRNA levels in the indicated cell lines. (**C**,**D**) qRT-PCR analyses of mRNA levels of ISG15, IL-6, CPT1, FABP4, and FASN in MDA-MB-468 cells. OE: LIPG-overexpressing MDA-MB-468 cells. KD: LIPG knockdown MDA-MB-468 cells. Control: MDA-MB-468 cells. (**E**) MDA-MB-231 cells were treated with dandelion extract for 24 h, and MTT assays were performed for cell viability. ** *p* < 0.01; *** *p* < 0.001. (**F**,**G**) Seahorse analyses of OCR in the indicated cell lines treated with dandelion (DAN) extract for 24 h.

To explore the mechanism by which LIPG promotes tumor cell metabolism, we stably transduced 468-WT cells with two different shRNAs that targeted LIPG (ST1 and ST2). To verify the reproducibility of our data, 468-WT cells were transfected with two siRNA LIPG (TS1 and TS2), and the qRT-PCR analysis confirmed that the shRNA or siRNA knockdown completely inhibits LIPG expression. Histone H3 lysine 27 acetylation (H3K27ac) is an essential mark of transcriptionally active loci [36]. A genome-wide analysis of H3K27ac occupancy by chromatin immunoprecipitation sequencing (ChIP-seq) revealed distinctive patterns of HDAC6 peaks in LIPG knockout cells compared to the parental counterparts (Figure 3A). We analyzed the active histone mark H3K27ac distribution across the transcriptional start site (TSS) (−/+5.0 Kb) genome-wide. High enrichment of H3K27ac was observed at TSS regions of histone deacetylase HDAC6 in 468-WT cells. Decreased H3K27ac peaks were observed within the HDAC6 locus in LIPG-depleted tumor cells. By combining RNA sequencing (RNA-seq) with ChIP-seq for active histone mark H3K27ac, we observed that a reduction in H3K27ac enrichment on HDAC6 promoter regions was associated with a reduction in HDAC6 gene expression in LIPG knockdown tumor cells (Figure 3B). These data suggest that LIPG modifies histone deacetylation and acetylation during lipid metabolism. These data also suggest that LIPG maintains HDAC6 mRNA expression through chromatin modifications.

Interferon-stimulated gene 15 (ISG15) is induced by type I interferon (IFN) and serves as an extracellular cytokine as well as an intracellular protein modifier [37]. IL-6 stimulates cancer cell proliferation, survival, and metastatic dissemination [38]. Additionally, IL-6 supports angiogenesis and tumor evasion of immune surveillance [39]. Given that LIPG mediates HDAC6 expression and histone H3K27ac in TNBC cells, we determined if LIPG may impact ISG15 and IL-6 gene expression in TNBC cells. A qRT-PCR analysis showed that LIPG overexpression in 468-OE cells increased the mRNA levels of ISG15 and IL-6 compared to the parent 468-WT (Figure 3C).

Tumor cells rely heavily on lipid metabolism, which supports the increased requirement for the synthesis of membranes and the activation of oncogenic signaling. Fatty acid synthase (FASN) is a key metabolic enzyme that dictates the terminal catalytic step in FA synthesis and promotes breast cancer metastasis [40]. An upregulation of FASN stimulates metastatic tumor cell growth, and FASN overexpression represents a hallmark of invasive tumor phenotypes in human malignancies [41,42]. Figure 3D shows that LIPG knockdown resulted in a marked downregulation of FASN mRNA expression in MDA-MB-468 cells.

Cytoplasmic fatty acid binding protein 4 (FABP4) induces insulin resistance by upregulating glucose production, and high circulating levels of FABP4 independently predict cardiometabolic risk and the risk of type 2 diabetes and breast cancer [43,44,45,46]. The mitochondrial fatty acid oxidation (FAO) pathway is activated in tumor cells with high energy demand. The rate-limiting enzyme CPT1 is associated with the outer mitochondrial membrane and regulates energy homeostasis since it is required for fatty acid β-oxidation in mitochondria [47,48]. We unexpectedly observed that LIPG knockdown slightly increased FABP4 mRNA but remarkably enhanced CPT1 mRNA expression. These data suggest that LIPG regulates tumor cell metabolism through chromatin modification.

### 3.7. Identification of a New LIPG Inhibitor, Cynaroside

Currently, there are no therapeutic LIPG inhibitors available for breast cancer patients. Motivated by our findings that support the role of LIPG in TNBC cell metabolism, we attempted to identify a new LIPG inhibitor for the potential treatment of TNBC. The herbal plant dandelion has been used as an antitumor agent in complementary medicine for thousands of years, and we previously reported that dandelion inhibits TNBC tumor cell growth [49]. Consistent with our previous finding, Figure 3E confirms that dandelion extract (20, 40, 80, and 160 μg/mL) in 1% FBS DMEM significantly inhibited MDA-MB-231 cell growth. Treatment with 100 μg/mL dandelion remarkably inhibited OCR in MCF-7 cells (Figure 3F) and suppressed the maximal respiration in MDA-MB-468 cells (Figure 3G). Thus, we decided to determine key the components of dandelion that are responsible for the inhibition of tumor cell metabolism.

**Figure 4:**
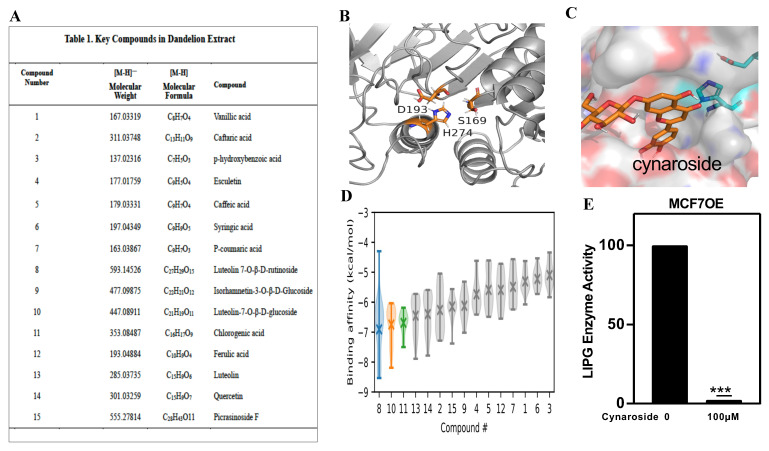
Identification of cynaroside as a new LIPG inhibitor. (**A**) UHPLC-ESI-Orbitrap MS/MS analysis was used to identify chemical components in dandelion extract [49]. The key components are summarized in the Table 1. (**B**) Zoomed-in view of the active-site binding pocket in LIPG. The catalytic triad (Ser169, Asp193, and His274) is shown in the stick representation. (**C**) Binding poses of luteolin-7-glucoside (cynaroside). (**D**) Estimated binding affinities of the 15 active compounds from the dandelion extract. The compound numbers (x axis) correspond to the compound numbers in (**A**). The binding affinities of 9 poses in 11 snapshots (taken from the last 100 ns of simulation) are shown in a violin plot. The median value is shown as a cross. Data for the top three compounds, 8 (luteolin 7-O-beta-rutinoside), 10 (cynaroside), and 11 (luteolin) are colored blue, orange, and green, respectively. (**E**) LIPG-overexpressing MCF7 cells (MCF7OE) were treated with cynaroside. The phospholipase activity of LIPG was analyzed for 20 min with 1 cycle/min as we previously described [10]. *** *p* < 0.001.

We used ultra-high performance liquid chromatography coupled with electrospray ionization hybrid linear trap quadrupole orbitrap mass spectrometry (UHPLC-ESI/LTQ-Orbitrap-MS) to isolate the active ingredients from the whole extraction of dandelion [49]. The active compounds from the dandelion extract are summarized in Figure 4A. To test which compounds can potentially inhibit LIPG, we first conducted a molecular modelling and docking study. A homology model of LIPG was built based on the recent X-ray structure of human lipoprotein, which shares the conserved active site, comprising a catalytic triad of Asp193, Ser169, and His274 (residue numbering in LIPG, Figure 4B). Following the molecular dynamics simulations to determine the protonation states and generate relaxed structures (Figure S3), docking was performed for the 15 active compounds in the binding pocket defined by the catalytic triad (Figure 4C and Figure S4). The binding affinities of the 15 compounds were estimated and ranked (Figure 4D and Figure S4). The top three compounds with decreasing affinities (i.e., the median value over various docked poses in LIPG conformations sampled from the molecular dynamics trajectory) were luteolin-7-O-beta-rutinoside, luteolin-7-glucoside (cynaroside), and chlorogenic acid (Figure 4D). While luteolin-7-O-beta-rutinoside had the highest median affinity, it had several high-energy poses. The docked compounds did not insert between the catalytic triad residues, but they made polar and hydrophobic contacts in the binding pocket.

**Figure 5:**
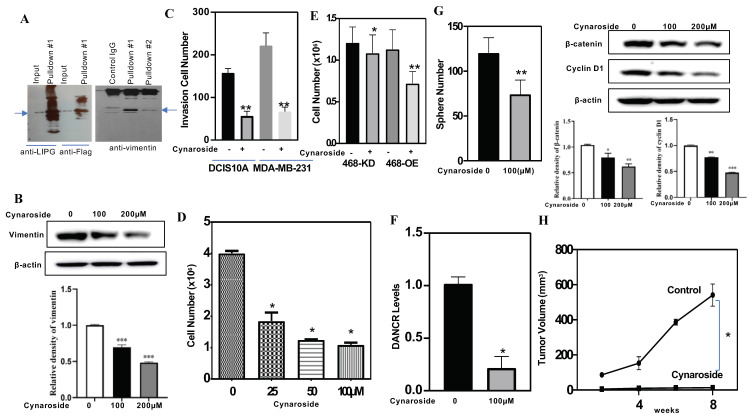
Verification of cynaroside antitumor activity in vitro and in vivo. (**A**) Pulldown assay for LIPG in MCF7OE cells. Anti-flag M2 antibody was used for LIPG pulldown and separated on 10% SDS-PAGE tris-glycine gel. Mouse vimentin antibody was used to detect vimentin. (**B**) MDA-MB-231 cells were treated with 100 μM and 200 μM cynaroside for 72 h. Western blotting was performed on whole-cell lysates. Blot quantification is shown below bands, and the uncropped blots are shown in Figure S6. (**C**) DCIS10A and MDA-MB-231 cells were treated with 100 μM cynaroside for 48 h, and a cell invasion assay was performed. (**D**) 468-OE cells were treated with different concentrations of cynaroside for 72 h. After treatment, cells were stained with trypan blue for cell counting. (**E**) 468-OE and 468-KD cells were treated with 100 μM cynaroside for 24 h, and cell number was counted. (**F**) qRT-PCR analysis of DANCR in 468-OE cells treated with 100 μM cynaroside for 72 h. (**G**) Left panel: 468-OE cells were harvested and then replated in the ultra-low attachment well containing the serum-free sphere culture medium with either vehicle or 100 μM cymaroside for sphere formation. After a week, the numbers of formed tumor-spheres were counted according to the sphere size criterion described [8,10]. Right panel: MDA-MB-231 cells were treated with cynaroside for 72 h, and Western blot was used to measure the proteins as indicated. Blot quantification is shown below bands, and the uncropped blots are shown in Figure S7. (**H**) Fourth mammary fat pads of 2-month-old female nude mice were transplanted with LIPG + IDC breast cancer cells as we previously described [8,10]. After tumor cell transplantation, mice were treated with either vehicle or cynaroside (60 mg/kg, *n* = 6) for 8 weeks. Animal tumor volumes were recorded and calculated. * *p* < 0.05; ** *p* < 0.01; *** *p* < 0.001.

To test if the top three compounds are LIPG inhibitors, MCF7OE cells were treated with 100 μM compounds. We selected cynaroside for the present studies because the LIPG enzyme assay showed that treatment with 100 μM cynaroside caused the nearly complete inhibition of LIPG enzyme activity in MCF7OE cells (Figure 4E) and prevented tumor growth (see Figure 5). These data indicate that cynaroside, a key component of dandelion, is a new LIPG inhibitor in breast cancer cells.

### 3.8. LIPG and Vimentin Interaction Facilitates Tumor Cell Invasion, and Cynaroside Inhibits LIPG and Vimentin to Impede Tumor Cell Invasion

TNBC cells express higher levels of vimentin, and vimentin knockdown impairs tumor cell migration [50]. Vimentin interacts directly with ubiquilin 2 (UBQLN2) and myotubularin-1 (MTM1), which promote post-translational modifications [51]. The glycosylation of vimentin is also important for vimentin-mediated cell migration [52,53]. To determine if LIPG interacts with vimentin, a flag-tag affinity procedure was used to purify an LIPG-containing complex, which was subjected to Western blot analysis. The physical interaction between LIPG and vimentin was confirmed by an immunoprecipitation (IP) analysis of MCF7OE cells. Purified LIPG bound directly to vimentin (Figure 5A). The IgG control showed undetectable signals. We performed an immunofluorescent analysis of vimentin and showed that an siRNA-mediated LIPG knockdown reduced vimentin expression in TNBC cells [8]. To further confirm our previous report, MDA-MB-231 cells were treated with 100 μM and 200 μM cynaroside for 72 h. Figure 5B shows that cynaroside downregulated vimentin. These findings suggest that the inhibition of LIPG by genetic and pharmacological approaches can lead to decreased vimentin in tumor cells. TNBC cells (DCIS10A and MDA-MB-231) were treated with cynaroside. A cell invasion assay showed that cynaroside significantly inhibited tumor cell invasion (Figure 5C). Thus, cynaroside could disrupt the interaction between LIPG and vimentin and subsequently reduced tumor cell invasion.

### 3.9. Cynaroside Inhibits Tumor Cell Growth In Vitro and In Vivo

A cell viability analysis was used to quantify living cells in cynaroside-treated 468-OE and 468-KD cells. 468-OE cells were treated with different concentrations of cynaroside (25, 50, and 100 μM) for 72 h. Figure 5D shows that cynaroside significantly inhibited cell growth in a dose-dependent manner. Treatment with 100 μM cynaroside significantly inhibited LIPG+ tumor cells (468-OE) and LIPG- tumor cells (468-KD) (Figure 5E), suggesting that cynaroside inhibited tumor cell growth through LIPG-dependent and LIPG-independent pathways.

Since Figure 1E demonstrates that DANCR binds to LIPG to maintain LIPG expression in TNBC cells, we decided to test if the inhibition of LIPG by cynaroside may impair DANCR. Treatment with cynaroside significantly inhibited DANCR expression in 468-OE cells (Figure 5F). The sphere formation assay is a method to measure the self-renewal capacity of cancer stem cells (CSCs). To examine the impact of cynaroside on the self-renewal of CSCs in LIPG-expressing TNBC, we performed sphere formation assays on 468-OE cells treated with cynaroside compared to control cells. We found that the total number of formed CSC spheres from 468-OE cells was significantly decreased by cynaroside treatment (Figure 5G, left panel). Wnt signaling is required for TNBC CSC self-renewal [54]. A Western blot analysis demonstrated that cynaroside inhibited β-catenin and cyclin D1 (an active marker of the β-catenin signaling pathway) in tumor cells (Figure 5G, right panel). These findings demonstrate that cynaroside attenuates the self-renewal of CSCs through the inhibition of Wnt signaling in tumor cells. Treatment with cynaroside remarkably inhibited OXPHOS in 468-OE cells (data not shown). These results suggest that targeting LIPG reprograms metabolisms to suppress tumor cell proliferation.

To test the effect of cynaroside on LIPG-expressing tumor development, we performed an in vivo tumorigenicity analysis on cynaroside treatment. As shown in Figure 5H, LIPG+ TNBC tumor cells were grown in nude mice. Treatment with cynaroside resulted in a significant inhibition of tumor growth in LIPG+ TNBC tumor cell xenograft models. These in vivo data strongly support that cynaroside suppresses TNBC tumorigenesis.

## 4. Discussion

Recently, there is a renewed attention on LIPG, which accompanies tumor initiation and growth in TNBC [7,8,9,10]; however, the mechanism of LIPG overexpression in tumor cells remains unclear. Previous studies revealed that a transcriptional factor, FoxA, and oxidative-stress-dependent AMPK contribute to the upregulation of LIPG in breast cancer cells [7,9]. DANCR is a cytoplasmic lncRNA that facilitates the functions of the oncogene network; our present data demonstrated that it directly binds to LIPG, providing stabilization in TNBC cells. Thus, our finding revealed a new mechanism by which DANCR regulates LIPG expression in tumor cells.

Metabolic reprogramming is a hallmark of TNBC progression, and little is known about the role of LIPG in the metabolic changes that accompany TNBC tumor growth. Here, we showed that TNBC cells exhibit a unique metabolic program characterized by LIPG-mediated OXPHOS. The silencing of LIPG by shRNA leads to a reduction in OXPHOS, suggesting that LIPG is a key regulator of metabolism in breast cancer. LIPG and DANCR knockdown lead to enhanced OA uptake. These data suggest that LIPG drives tumor cell growth through a bifurcation of metabolic programs between OXPHOS and FA metabolism, leading to the discrepancy between these LIPG-positive and LIPG- negative tumors. A ChIP-seq analysis showed a clear decrease in H3K27ac at the HDAC6 locus in LIPG knockdown tumor cell models, indicating that LIPG epigenetically mediates tumor cell metabolism through chromatin modifications.

Our previous reports demonstrated that LIPG activates cancer stem cell self-renewal and promotes TNBC tumorigenesis in vivo [8,10]. Based on these findings, we speculated that targeting LIPG is a promising strategy in cancer therapy. Currently, a few synthetic LIPG inhibitors have been reported with inhibition efficiency in cell-free LIPG enzyme assays [55,56]; however, these inhibitors cannot be used to treat breast cancer due to poor permeability in tumor cells. In this study, we showed that cynaroside isolated from the antitumor herbal plant dandelion has a remarkable LIPG-inhibitory profile. Cynaroside exerts anti-proliferative activity against TNBC tumor cells and inhibits cancer stem cell self-renewal as well as OXPHOS. Furthermore, cynaroside shows antitumor activity in LIPG+ breast cancer cell xenograft models. These data suggest that the treatment of breast cancer cells with cynaroside has the potential to prevent tumor formation and eliminate latent tumor cell metastasis.

## 5. Conclusions

LIPG knockdown by siRANs or shRANs led to metabolic changes characterized by a reduction in mitochondrial OXPHOS and a concomitant increase in oleic acid uptake in tumor cells. The importance of LIPG for tumor cell mitochondrial metabolism prompted us to test the therapeutic potential of LIPG inhibitors. Towards this end, we discovered a natural compound, the LIPG inhibitor cynaroside, which efficiently suppressed LIPG enzyme activity as well as OXPHOS in TNBC cells, leading to impaired tumor formation in vivo. Taken together, our work demonstrates that developing LIPG inhibitors is a promising new therapeutic approach for the treatment of breast cancer.

## Data Availability

The data presented in this study are available in this article.

## Supplementary Materials

The following supporting information can be downloaded at: https://www.mdpi.com/article/10.3390/cancers14153763/s1, Figure S1: DANCR binds to LIPG in LIPG-overexpressing MCF-7 cells, Figure S2: Identification of a specific DANCR-binding region for LIPG, Figures S3 and S4: Molecular docking for the active compounds from the dandelion extract, Figures S5–S7: Full pictures of the Western blots.
