# Supplementary material for: A Proof-of-Concept Inhibitor of Endothelial Lipase Suppresses Triple-Negative Breast Cancer Cells by Hijacking the Mitochondrial Function
Source: Cancers (Basel). 2022 Aug 2;14(15):3763. doi: 10.3390/cancers14153763 (PMC9367514; doi:10.3390/cancers14153763)
Supplement: Supplementary file 1 [file cancers-14-03763-s001.zip › Suppemental Materials and Data_Final.pdf]

Supplementary Data

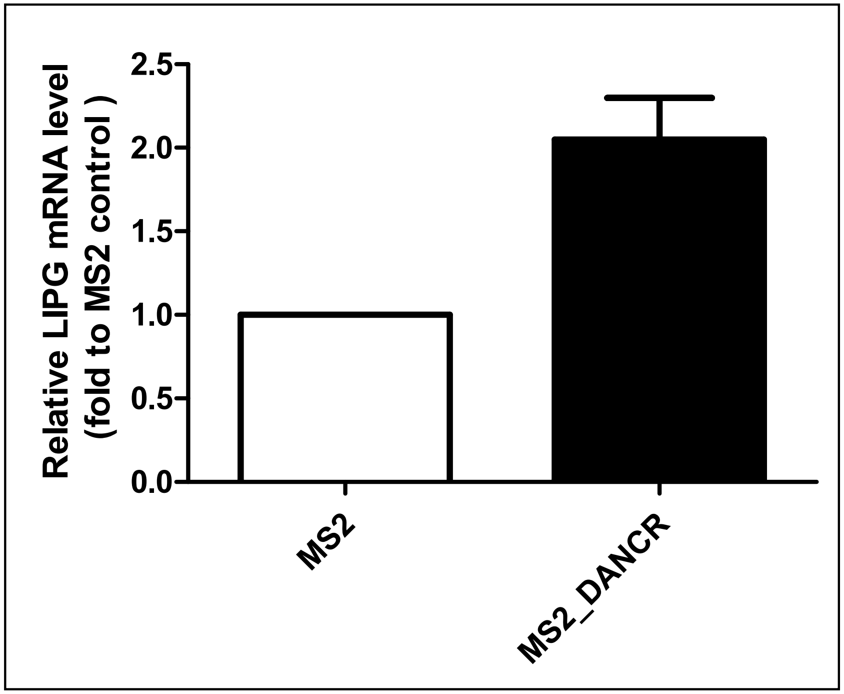

Figure S1. LIPG stable overexpressing MCF7 cells were co-transfected with p_NLS_MCP_GFP and pCDNA3-MS2 (negative control) or pCDNA3-MS2_hDANCR. Cells were collected 48 hours after transfection for RNA pulldown assay. A qRT-PCR analysis was performed to measure LIPG mRNA levels.

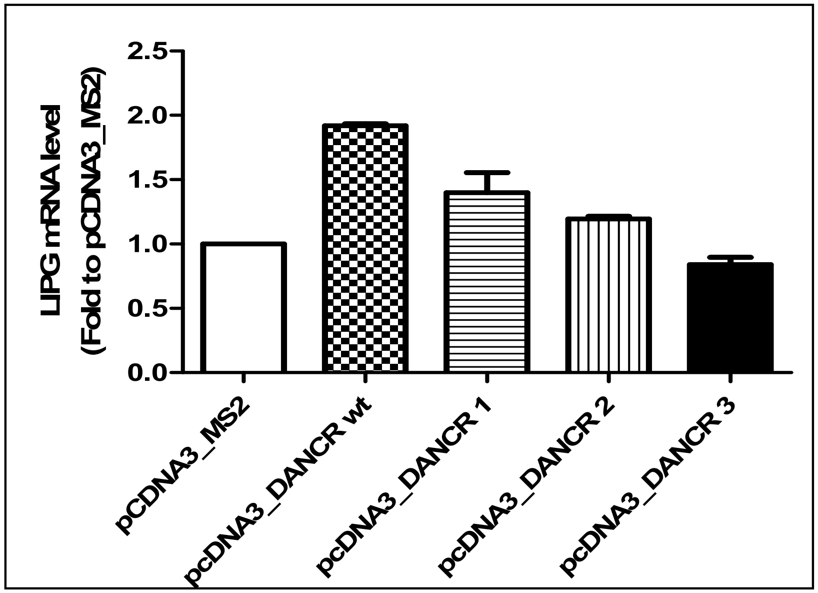

Figure S2. LIPG stable overexpressing MCF7 cells were co-transfected with p_NLS_MCP_GFP and pCDNA3-MS2 (negative control) or different length of pCDNA3-MS2_hDANCR. Cells were collected 48 hours after transfection for RNA pulldown assay. A qRT-PCR analysis was performed to measure LIPG mRNA levels.

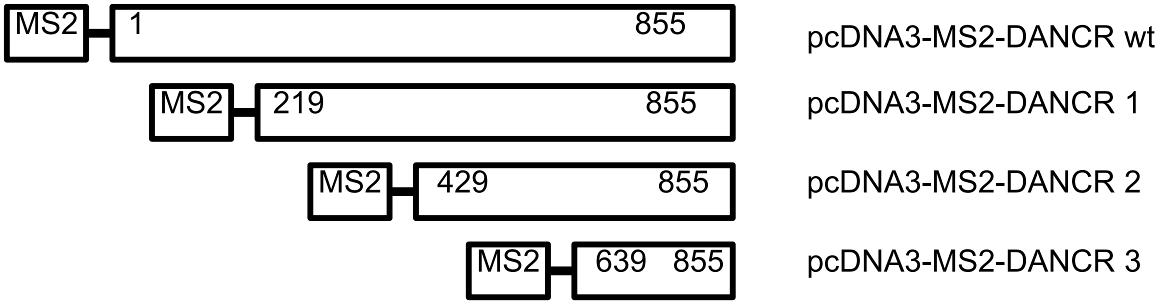

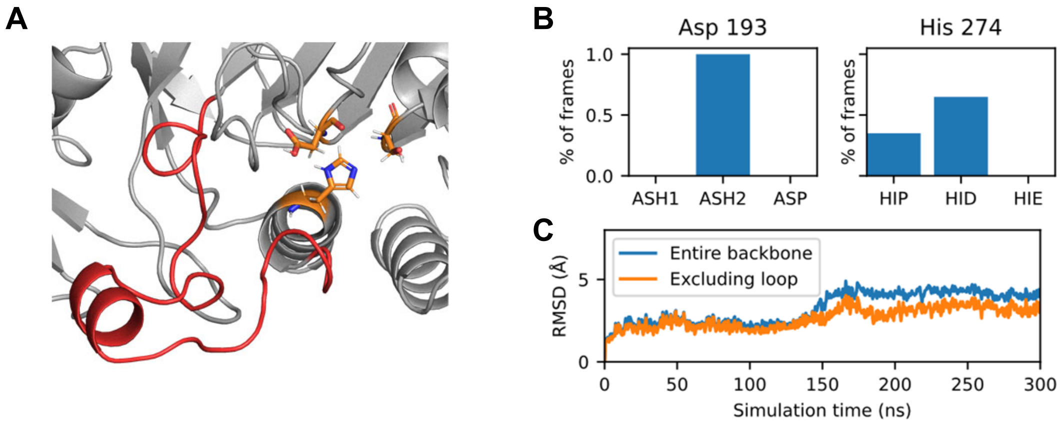

Figure S3: (**A**) Zoomed-in view of the binding pocket, with the catalytic triad (Ser169, Asp193, and His274) highlighted in orange and a nearby 29-residue dynamic loop in red. (**B**) Predicted protonation/tautomer state populations for Asp193 (protonated) and His274 (predominantly singly protonated as HID) from the continuous constant pH molecular dynamics simulations. (**C**) Root mean square deviation of the backbone atoms before (blue) and after (orange) removing the flexible loop near the binding site over a 300-ns conventional molecular dynamics simulation.

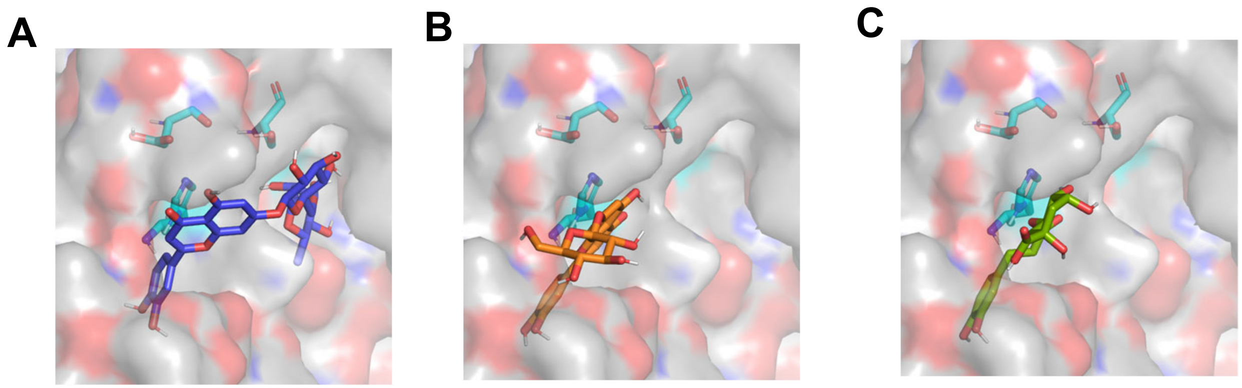

Figure S4: Binding poses of the top three compounds with the highest binding affinities: luteolin 7-O-beta-rutinoside (**A**), cynaroside (**B**), and luteolin (**C**). The catalytic triad Ser169, Asp193, and His274 highlighted as cyan sticks.

Figure S5

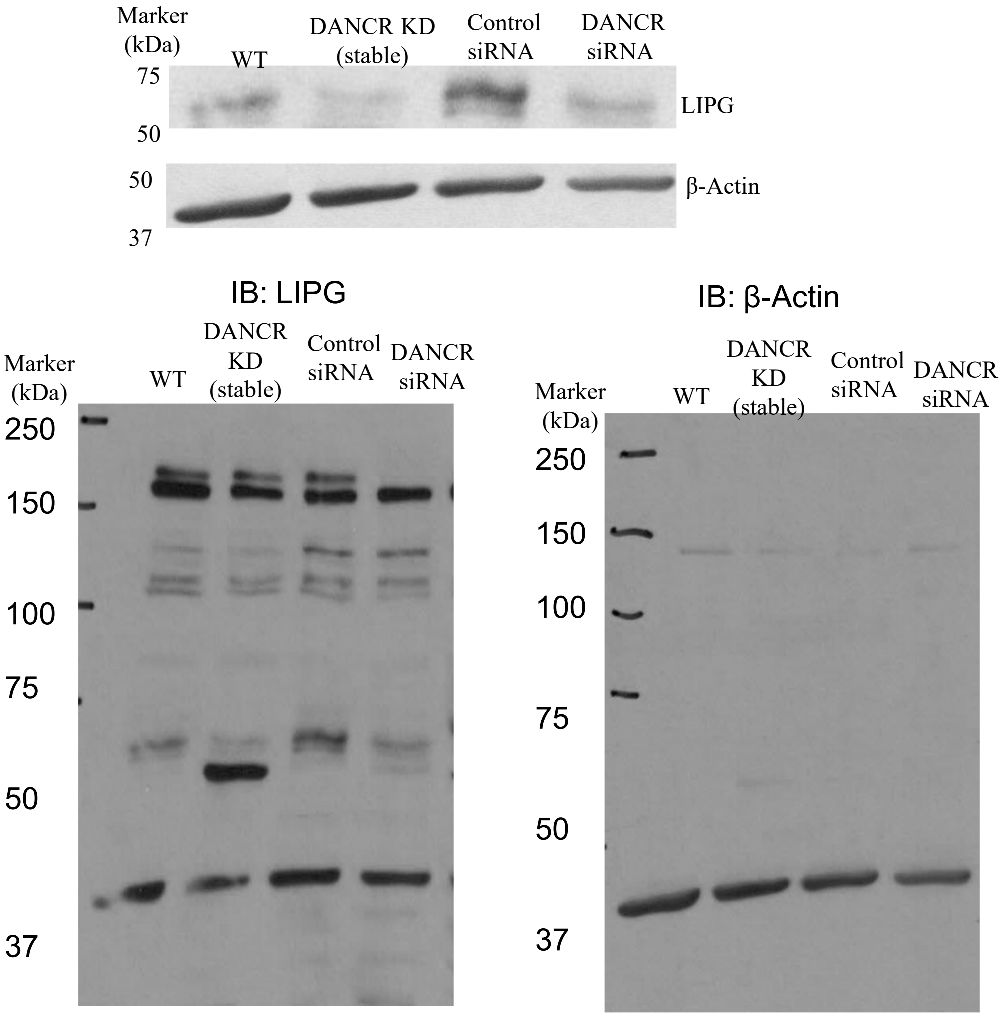
Marker (kDa)
WT
DANCR KD (stable)
Control siRNA
DANCR siRNA
75
50
LIPG
50
37
β-Actin
IB: LIPG
Marker (kDa)
WT
DANCR KD (stable)
Control siRNA
DANCR siRNA
250
150
100
75
50
37
IB: β-Actin
Marker (kDa)
WT
DANCR KD (stable)
Control siRNA
DANCR siRNA
250
150
100
75
50
37

Figure S6

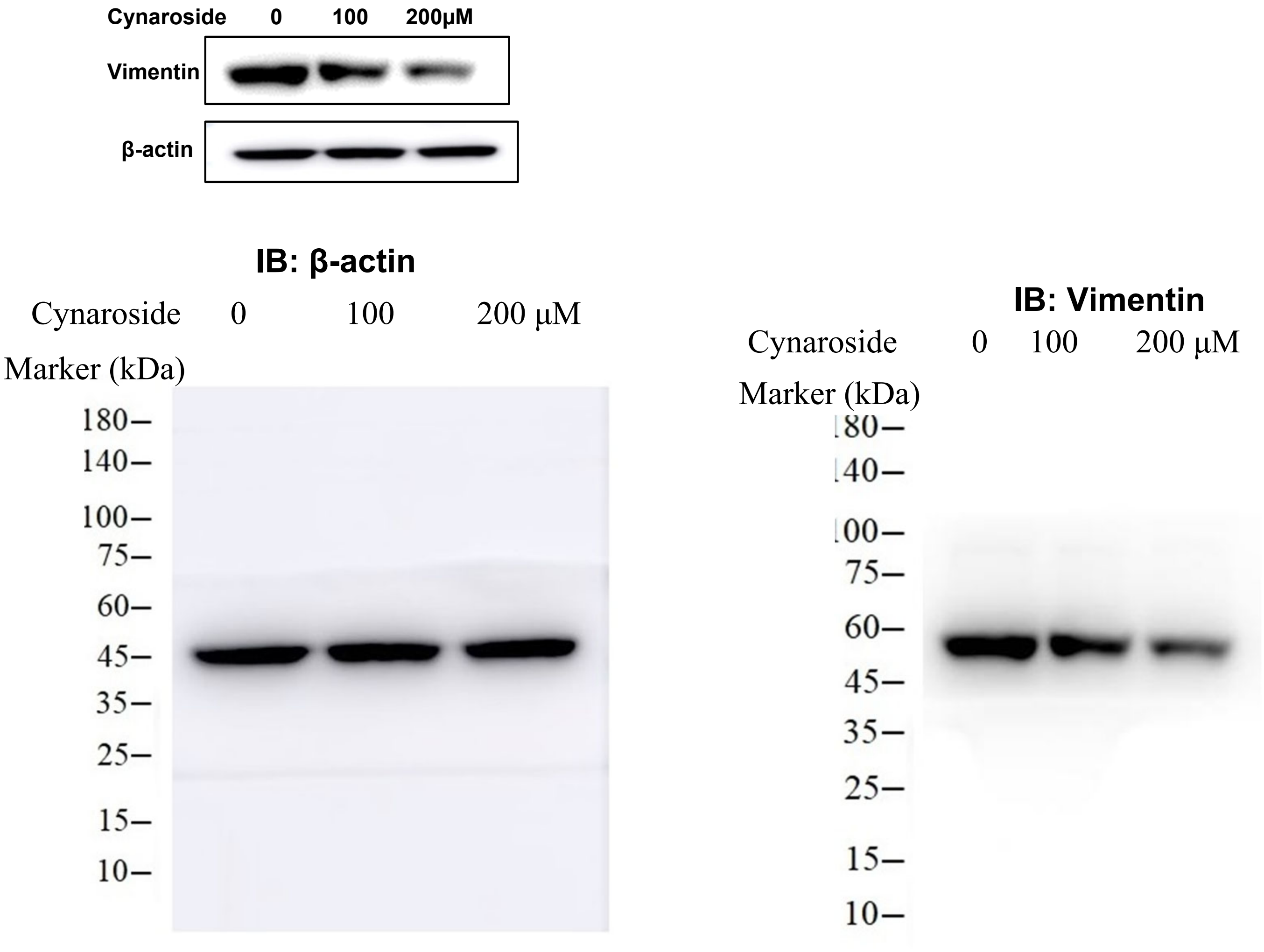
Cynaroside 0 100 200μM
Vimentin
β-actin
IB: β-actin
Cynaroside 0 100 200 μM
Marker (kDa)
180
140
100
75
60
45
35
25
15
10
IB: Vimentin
Cynaroside 0 100 200 μM
Marker (kDa)
75
60
45
35
25
15
10

Figure S7

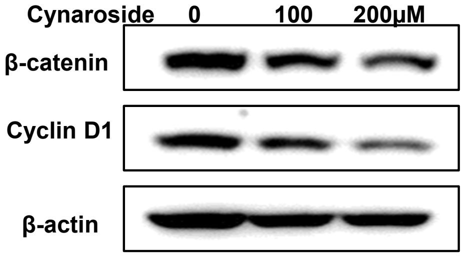
Cynaroside 0 100 200μM
β-catenin
Cyclin D1
β-actin

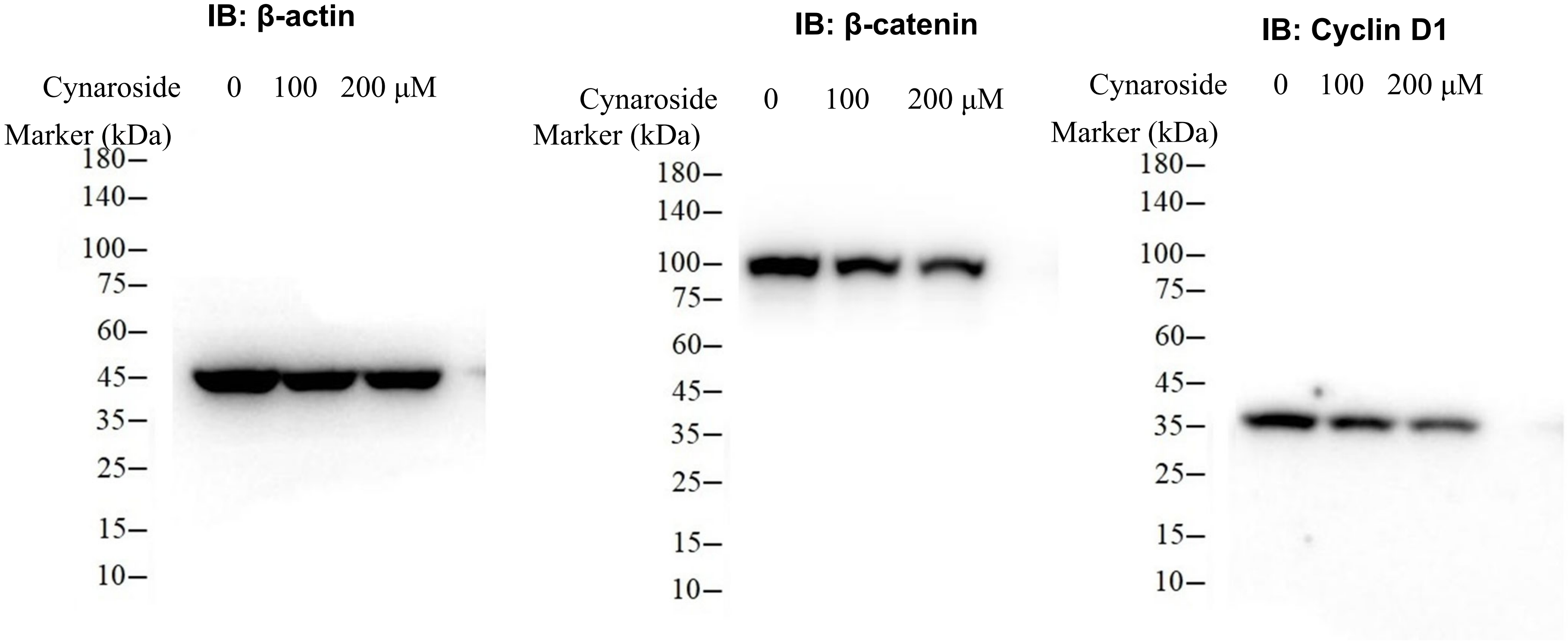
IB: β-actin
Cynaroside 0 100 200 μM
Marker (kDa)
180
140
100
75
60
45
35
25
15
10
IB: β-catenin
Cynaroside 0 100 200 μM
Marker (kDa)
180
140
100
75
60
45
35
25
15
10
IB: Cyclin D1
Cynaroside 0 100 200 μM
Marker (kDa)
180
140
100
75
60
45
35
25
15
10
